# Incidence and predictors of immediate complications following perioperative non-obstetric epidural punctures

**DOI:** 10.1186/1471-2253-12-31

**Published:** 2012-12-10

**Authors:** Andreas Meyer-Bender, Andrea Kern, Bernhard Pollwein, Alexander Crispin, Philip M Lang

**Affiliations:** 1 Department of Anaesthesiology, University Hospital of Munich, Marchioninistr. 15, 81377, Munich, Germany; 2 Department of Medical Informatics, Biometry, and Epidemiology, Ludwig Maximilians University Munich, Munich, Germany

## Abstract

**Background:**

Epidural Anesthesia (EA) is a well-established procedure. The aim of the present study was to evaluate the incidence of immediate complications following epidural puncture, such as sanguineous puncture, accidental dural perforation, unsuccessful catheter placement or insufficient analgesia and to identify patient and maneuver related risk factors.

**Methods:**

A total of 7958 non-obstetrical EA were analyzed. The risk of each complication was calculated according to the preconditions and the level of puncture. For probabilistic evaluation we used a logistic regression model with forward selection.

**Results:**

The risk of sanguineous puncture (n = 247, 3.1%) increases with both the patient’s age (P = 0.013) and the more caudal the approach (P < 0.01). Dural perforation (n = 123, 1.6%) was found to be influenced only by advanced age (P = 0.019). Unsuccessful catheter placement (n = 68, 0.94%) occurred more often in smaller individuals (P < 0.001) and at lower lumbar sites (P < 0.01). Amongst all cases with successful catheter placement a (partial) insufficient analgesia was found in 692 cases (8.8%). This risk of insufficient analgesia decreased with patient’s age (P <0 .01), being least likely for punctures of the lower thoracic spine (P < 0.001).

**Conclusions:**

Compared to more cranial levels, EA of the lower spine is associated with an increased risk of sanguineous and unsuccessful puncture. Insufficient analgesia more often accompanies high thoracic and low lumbar approaches. The risk of a sanguineous puncture increases in elderly patients. Gender, weight and body mass index seem to have no influence on the investigated complications.

## Background

Due to its advantageous benefit-to-risk profile epidural anesthesia (EA) is a well-established procedure in contemporary anesthesia [1]. EA is an inherent part of fast-track concepts [2-7] and reduces the perioperative hazard in high risk patients [8-10]. Fortunately, serious long-term complications are very rare [11-14], but immediate complications or failures of the method are frequently seen in the operating room. The overall incidence of sanguineous puncture is between 0.7 and 15.7% [15-19], but decreases with more cranial approaches [15]. Accidental dural puncture occurs in up to 2.7% of EA procedures [14,15,17,19,20], again more often in lower spinal levels [14,15]. Unsuccessful catheter placement was described in 0.13 to 4.6% [14-16,21] while insufficient analgesia is seen in up to 12% [15-17,19,21]. To date, specific risk factors associated with acute complications like sanguineous puncture, dural perforation, insufficient analgesia and inability to place the catheter have not been extensively examined. Accordingly, this study set out to investigate the influence of patient’s age, gender, biometrics or the chosen spinal level on acute complications associated with EA. A retrospective approach was taken using a database of electronic anesthesia reports of 7958 adult patients who received perioperative non-obstetric EA between 2004 and 2009.

## Methods

In our institution, epidural anesthesia (EA, stand-alone or combined with a general anesthesia) is the preferred anesthetic procedure for most major abdominal, thoracic, vascular, urologic, gynecological and orthopedic surgery. EA is administered at all spinal levels from TH2/3 to L5/S1. A midline approach in the sitting position using the “loss-of-resistance” technique with sterile saline is the standard procedure. In cases of combination with general anesthesia, EA is loaded with a bolus opioid and local anesthetics immediately after induction of general anesthesia. While intraoperative analgesia is maintained with intermittent bolus application of local anesthetics, every epidural catheter is connected postoperatively with either a continuous running or a patient controlled infusion pump containing a local anesthetic and an opioid.

### Data collection and extraction

An electronic anesthesia information management system (AIMS) runs in most operating divisions. After establishing an EA, the anesthesiologist is obligated to document the procedure in the AIMS, including mandatory fields for chosen spinal level and needle type, distance between skin and epidural space, depth of the inserted catheter, and success of the procedure. In addition, fields for a free text statement and check boxes for sanguineous or dural puncture are available. Completed anesthetic reports are stored both on a file server for fast access and in a relational database (Oracle®, Oracle Corp., Redwood Shores CA), suitable for administrative or scientific analysis.

After approval by the local ethics committee of the Ludwig-Maximilians-University Munich (Marchioninistr. 15, 81377 Munich, Germany) we performed a query within this database using MSQuery® (Microsoft® Corp., Redmond WA). Initially, for each patient aged 18 years or above and having received an EA between 2004 and 2009 values of age, gender, height, weight and body mass index the site of puncture and its success were extracted from the database. In a second step, the data from the recovery room were surveyed to identify all patients with insufficient analgesia. For this procedure, we considered the analgesic effect of the EA as (partial) insufficient when an intravenous patient-controlled analgesia system was administered or the postoperative dose of piritramide was more than 10 mg. All data were collected without personal information. The two data sets were matched with Excel 2007® (Microsoft Corp., Redmond WA) using the unique ID number of each anesthetic report. In order to achieve irreversible anonymity this ID number was erased from the data sheet after plausibility control before the statistical analysis was performed.

### Statistical analysis

The analysis of the validated data was performed using SAS 9.2 for Linux (SAS Institute, Carry NC). Analysis was carried out using logistic regression models with forward selection at an alpha level of 5%. The spinal segments were grouped into six anatomical regions. The lower thoracic spine (TH9-11) - representing the largest fraction - acted as reference for all other levels.

## Results

From an initial 8101 data sets (7614 EA, 487 combined spinal epidurals (CSE)) 141 EA and 2 CSE were excluded, leaving 7958 data sets for the study. In 26 cases data input of weight or height were obviously in error and thus were excluded. In 39 reports the standard approach was abandoned (9 single shot caudal blocks, 30 paramedian/lateral punctures). 78 doublets had to be consolidated due to the fact that more than one EA had been recorded to describe several attempts in one patient.

54.6% of the patients were male (n = 4342), 45.4% female (n = 3616). They had a mean age of 64 years (median 60.7, min-max 18-103), a mean BMI of 26.2 kg×cm^-2^ (median 25.7, min-max 11.7-67.9) and a mean height of 170 cm (median 170, min-max 117-204). Distributions of descriptive characteristics for the patient population are shown in Table 1. The majority of the study population comprised middle- and south-European caucasians.

**Table 1:** Distribution of the patient’s age, body mass index (BMI) and height

**Age**	**n**	**BMI**	**n**	**Height**	**n**
**[years]**	**[%]**	**[kg×cm**^**-2**^**]**	**[%]**	**[cm]**	**[%]**
18-20	62 (0,8)	0- 18,5	262 (3,3)	0- 140	7 (0,1)
21 - 30	302 (3,8)	19 - 25	3304 (41,5)	141 - 150	132 (1,7)
31 - 40	463 (5,8)	26 - 30	2873 (36,1)	151 - 160	1234 (15,5)
41 - 50	963 (12,1)	31 - 35	1099 (13,8)	161 - 170	2820 (35,4)
51 - 60	1544 (19,4)	36 - 40	285 (3,6)	171 - 180	2787 (35)
61 - 70	2566 (32,2)	41 - 45	87 (1,1)	181 - 190	881 (11,1)
71 - 80	1606 (20,2)	46 - 50	31 (0,4)	191 - 200	95 (1,2)
81 - 90	414 (5,2)	51 - 55	9 (0,1)	201 - 210	2 (0)
91 - 100	37 (0,5)	56 - 70	8 (0,1)		
101 - 110	1 (0)				

Data are presented as absolute numbers (n) and in relationship to the whole patient population investigated [%].

**Figure 1:**
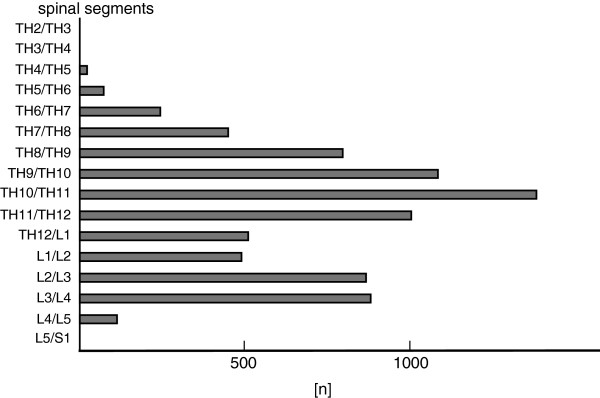
**Illustrated is the distribution of the approached spinal levels.** The low-thoracic approach was preferred by establishing an EDA. The high thoracic approach was done in a rare manner.

The distribution of segment chosen was bimodal with middle lumbar and lower thoracic maxima (illustrated in Figure 1).

A mean of 1.34 attempts (1 – 9, min – max) were necessary to place the epidural catheter.

Logistic regression of level punctured and the consecutive immediate complications are shown in Table 2.

**Table 2:** Logistic regression of level punctured and immediate complications

	**Sanguineous puncture (P=0.0054)**			**Unsuccesful catheter placement (P=0.0024)**			**Insufficient analgesia (P=0.0001)**		
**Parameter**	**Odds Ratio**	**95%-CI**		**Odds Ratio**	**95%-CI**		**Odds Ratio**	**95%-CI**	
**TH2/3 - TH6/7**	0.963	0.442	2.096	1.762	0.340	9.132	1.954	1.326	2.881
**TH7/8 - TH8/9**	0.862	0.558	1.333	2.887	1.094	7.617	1.696	1.318	2.181
**TH11/12 - TH12/L1**	1.369	0.886	2.116	1.517	0.495	4.653	1.243	0.936	1.651
**L1/2 - L2/3**	1.586	1.031	2.440	2.436	0.843	7.037	1.240	0.924	1.663
**L3/4 - L5/S1**	1.716	1.092	2.696	5.753	2.138	15.478	1.553	1.145	2.105

Reference level for the odds ratio is TH9-11. Odds-Ratio and 95%-Confidence interval are given.

### Sanguineous puncture

In 247 cases, representing 3.1% of all attempts, a sanguineous puncture (SP) was reported. Age and the spinal segment chosen were identified as independent risk indicators for SP, whereas height and BMI were not correlated with SP incidence. Risk of SP increased with an odds ratio (OR) of 1.012 (P = 0.01; CI: 1.003 – 1.022) with each year of age. Lumbar approaches resulted in higher SP rates than thoracic punctures (Table 2).

### Accidental dural perforation

Perforation of the dura mater was seen in 123 cases (1.6% of all cases). Dural puncture was more frequent in older patients (OR = 1.017 per year, CI 1.004-1.031, P = 0.01) and neither spinal level, gender, height nor BMI showed influence on its occurrence.

### Unsuccessful catheter placement

Puncture of the epidural space was aborted without success in 68 cases (0.9%). The risk of unsuccessful catheter placement (UCP) decreased with increasing body height (OR = 0.952 per cm, CI 0.928-0.976, P < 0.001) and was influenced by the level punctured (P = 0.0024). The lower lumbar (OR = 5.753, CI 2.138-15.478, P < 0.001) and the middle thoracic spine (OR = 2.887, CI 1.094-7.617, P = 0.03) both showed an elevated risk of UCP compared to the reference region TH9-11 (Tab. 2).

### Insufficient analgesia

In 692 of 7890 successful punctures either the analgesia was rated insufficient by the anesthesiologist in the course of surgery or the patient received increased amounts of intravenous opiates in the recovery room (8.8%). The risk of insufficient analgesia (IA) decreased with patient age. The incidence of insufficient analgesia was higher in the upper thoracic and lower lumbar spine when compared to the reference level (TH9-11; Tab. 2).

**Figure 2:**
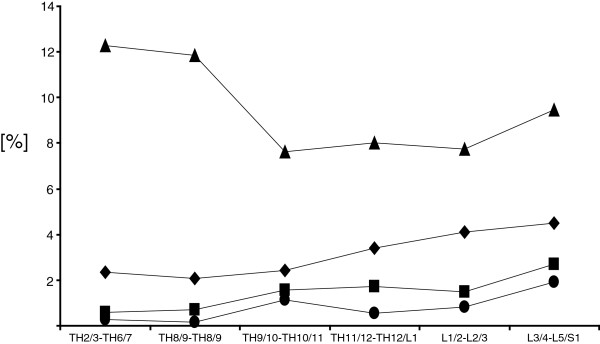
**Incidences of complications in relation to punctured spinal region are illustrated.** Triangle: insufficient analgesia: high incidence in high- and mid- thoracic area; diamond: sanguineous puncture, square: accidental dural puncture; circle: unsuccessful catheter placement.

**Figure 3:**
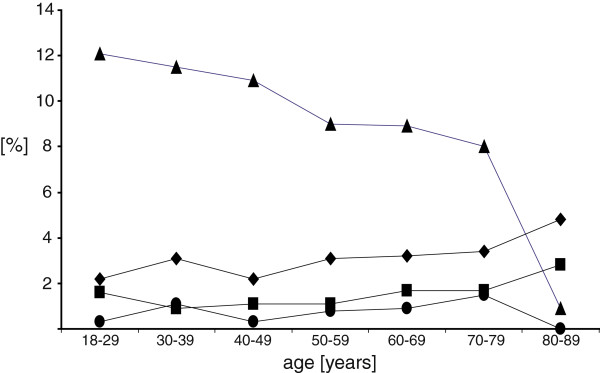
**Incidences of complications in relation to patient’s age are shown.** Triangle: insufficient analgesia is decreasing with higher age; diamond: sanguineous puncture; square: accidental dural puncture; circle: unsuccessful catheter placement.

Figures 2 and 3 illustrate the incidences of the above mentioned complications in relation to age and punctured spinal level.

### Paresthesia

Transient paresthesias were reported in 223 (2.8%) punctures For these cases however, neither persistent paresthesia nor any other serious neurologic event were reported. The risk of paresthesia was reduced in patients with advanced age (OR = 0.990; CI: 0.981 – 0.998, P < 0.01) and in males (OR = 0,665; CI: 0.507 – 0.872, P = 0.02).

## Discussion

Our data indicate that advanced age was associated with a higher risk of sanguineous and dural puncture, although advanced age was also associated with a lower incidence of insufficient analgesia. The level chosen for epidural puncture influenced the rate of sanguineous puncture, unsuccessful catheter placement and insufficient analgesia. Height was the only anthropometric index correlated with immediate complications, the risk of unsuccessful catheter placement being less in taller individuals.

### Sanguineous puncture

The incidence of sanguineous puncture (SP) has previously been reported at between 0.7 and 15.7% [15-19]. A recent meta-analysis comparing air and liquid as a medium for loss of resistance found an overall incidence of 5.9% in parturients [21]. Most of the published data relates to obstetric EA and considering that the probability of complications is estimated to be higher in pregnancy (3 to 15.7% in obstetric EA [16-19] vs. 0.7 in EA for lower abdominal procedures [15]), our finding of 4.4% while broadly consistent with previous reports is somewhat higher than in non-obstetric EA.

Our findings of more SP in the lumbar spine may be due to the anatomy of the posterior venous spinal plexus. This plexus occupies a considerably larger volume in the lumbar area than further cranial [22]. Moreover, this venous system has no valves, which causes an additional distension of the caudal vascular bed when the patient is in an upright position [23]. An analysis investigating potential factors reducing the risk of intravascular catheter placement demonstrated that the lateral position was beneficial (3.7% vs. 15.7%) [24]. In addition, a thoracic approach seems to reduce the risk of SP [15] and this finding is consistent with our data.

Neither the risk of SP in elderly patients nor age-based changes of the intraspinal venous plexus have been previously evaluated systematically. Therefore, we can only speculate as to whether varicose changes and/or a higher vulnerability in older people may contribute to the higher incidence of SP in our study population.

### Accidental dural perforation (ADP)

A rate of 1.6% ADP is in accordance with data published previously (0% and to 2.7% [14,15,17,19,20]. Two studies found a higher incidence of ADP in lower spinal levels – one study evaluated only the thoracic region [14], while the other study investigated the entire spine [15]. Our results show similar trends, with a higher incidence of ADP in the lumbar spine. An explanation for this observation could be related to the reduction in thickness of dura mater around lumbar level L2/3 [25]. To our knowledge, a more frequent occurrence of ADP in older patients has not been described before. A potential hypothesis to explain this might be that typical age-related changes like degenerative disc disease, facet joint arthritis and progressive kyphosis may make the identification of the epidural space more difficult than in younger patients. Furthermore, stenosis of the spinal canal, a typical disease of the elderly, might result in adhesive processes which could reduce the epidural space [26]. In summary, these factors may explain the higher incidence of ADP.

### Unsuccessful catheter placement (UCP)

The incidence of unsuccessful catheter placement (0.9%) is in agreement with previously published data: among obstetric patients the incidence of abandoned trials to establish EA was estimated between 0.5% to 4.6% [19,21], among non-obstetric patients 0.13% [15] and 1.1% for thoracic EDA [14]. Previous studies did not detect any relation between level and UCP incidence [15]. In a regression analysis of our data, a correlation was found between punctured level and UCP: the lower lumbar and middle thoracic spine were associated with a discernably higher risk than punctures at the reference level (TH9-11).

### Inadequate analgesia (IA)

The observed incidence (8.8%) (at least partially) insufficient EA is commensurate with published data (0% to 12% [15-17,19,21]. The wide range of IA reported in the literature might be due to the absence of a commonly accepted definition of insufficient analgesia. Some authors claim a failure of the method only when an unscheduled additional general anesthesia was required [17,19], while others use the visual analog scale to measure pain intensity [16]. Harney et al. classified an asymmetric effect as failure to insert [19]. Our concept, to classify EA as insufficient on the basis of the postoperative opioid requirement may have led to our relatively elevated incidence of IA.

The regional distribution of IA is remarkable in that we observed a higher incidence in the high thoracic and the low lumbar regions. Our presumption is that in many of these cases the catheter was placed correctly but the local anesthetic did not reach all spinal segments needed for analgesia of the entire site of operation. Therefore choosing a very high or low spinal region for epidural puncture may lead to analgesic insufficiency.

### Paresthesia

The risk of paresthesia is lower in both male patients and those of advanced age. The overall incidence of 2.8% is lower compared to data acquired from spinal anesthesia (6.3% [27]). However, reports of transient paresthesia are a rare feature potentially influenced by subjective bias of the anesthesiologist in reporting such events. In the current study, reports of persistent paresthesias were not encountered.

### Technical considerations

The appropriate identification of the level punctured during EA and the experience and subjective bias of anesthesiologists may have considerable impact on the quality of the data collected and therefore these features need to be controlled for.

The spinal level is usually identified on the basis of imprecise landmarks and there are certainly a number of cases where the punctured segment is not identified correctly. To reduce systematic error associated with misjudged level the technique of grouping segments by anatomical area was used [15].

It is more difficult to account for differences in the expertise of the anesthesiologists performing EA. One might assume that most trainees gain first EA experience with lumbar punctures due to the absence of the spinal cord in this area. This could be a reason for some complications occurring more often in caudal areas and explain similar findings from other publications. However, in the case of inability to place the catheter, every trainee of our department is required to consult a senior consultant before aborting the procedure of EA. Therefore, only experts are responsible for the higher incidence of UCP in the lumbar area. The higher incidence of sanguineous puncture can be accounted for anatomic reasons and is rather unlikely to be related to the anesthesiologist’s experience. Whether training of the anesthesiologist influences the rate of accidental dural puncture cannot be retrospectively examined using our data because the data are anonymized and the anesthesiologist who performed the epidural puncture cannot be directly identified.

## Conclusion

The present dataset comprising 7958 EA demonstrated that EA of the lower spine is associated with a higher risk of sanguineous and unsuccessful puncture. Insufficient analgesia was seen more frequently in high thoracic and low lumbar approaches. The risk of sanguineous and dural puncture increases in elderly patients. While unsuccessful epidural approaches occur more often in smaller individuals, gender, weight and body mass index were not correlated with the complications named above. In general, immediate complications following EA occurred relatively infrequently. Nevertheless, being aware of certain populations potentially at higher risk of complications might further improve the safety in performing EA.

## Competing interests

All authors declare they have no competing interests.

## Authors’ contributions

AMB and PML conceived the study. PML designed the study. BP an AMB were responsible for data extraction. AK, AC and AMB analyzed the data. AC performed the statistical analysis. All authors participated in study design and the conduct of study. AMB and PML were responsible for data analysis and manuscript preparation. All authors reviewed and approved the final manuscript.

## Pre-publication history

The pre-publication history for this paper can be accessed here:

http://www.biomedcentral.com/1471-2253/12/31/prepub
